# Limited efficacy of 3 + 7 plus gemtuzumab ozogamycin in newly diagnosed fit intermediate genetic risk acute myeloid leukemia patients

**DOI:** 10.1002/cnr2.2044

**Published:** 2024-04-25

**Authors:** Bianca Serio, Francesco Grimaldi, Lucia Ammirati, Mario Annunziata, Giovanna De Santis, Alessandra Perrotta, Danilo De Novellis, Valentina Giudice, Denise Morini, Gabriella Storti, Catello Califano, Antonio Maria Risitano, Fabrizio Pane, Carmine Selleri

**Affiliations:** ^1^ Hematology Unit University Hospital “San Giovanni di Dio e Ruggi d'Aragona” Salerno Italy; ^2^ Hematology Unit, Department of Medicine and Surgery University of Naples “Federico II” Naples Italy; ^3^ Hematology Unit, Hospital “A. Tortora” Pagani Italy; ^4^ Hematology Unit, Hospital “S. G. Moscati” Aversa Italy; ^5^ Hematology Unit, Hospital “S. Giuseppe Moscati” Avellino Italy; ^6^ Department of Medicine, Surgery, and Dentistry University of Salerno Baronissi Italy

**Keywords:** acute myeloid leukemia, chemotherapy, efficacy, safety

## Abstract

**Background:**

Gemtuzumab‐ozogamycin (GO) is approved in combination with high‐dose chemotherapy for treatment‐naïve low‐ and intermediate‐risk acute myeloid leukemia (AML).

**Aims:**

In this retrospective real‐life multicenter study, we reported efficacy and safety of GO plus high‐dose chemotherapy in newly diagnosed AML patients.

**Methods and Results:**

A total of 31 fit low‐ and intermediate‐risk AML patients treated with GO‐based regimens were retrospectively included in this real‐life multicenter study, and results were compared with a control cohort treated with 3 + 7 alone. Complete remission (CR) rate after induction was 77%, and most responders (45%) underwent two GO‐based consolidation, and minimal residual disease (MRD) negativity was observed in 17 cases (55%) after the end of consolidation. Low genetic risk was associated with increased CR rate compared with intermediate‐risk AML (88% vs. 33%; *p* < .001), as well as prolonged overall survival (OS; hazard ratio, 0.16; 95% confidential interval, 0.02–0.89; *p* < .001). GO addition resulted in a survival benefit for low‐risk AML (median OS not reached vs. 25 months; *p* = .19) while not for intermediate‐risk subjects (10 vs. 13 months; *p* = .92), compared with the control group. Moreover, GO‐treated patients experienced fever of unknown origin or sepsis in 42% or 36% of cases, respectively, with one death during induction due to septic shock, with similar rates compared with the control group (*p* = .3480 and *p* = .5297, respectively). No cases of veno‐occlusive disease after allogeneic transplantation were observed.

**Conclusions:**

Our real‐life multicenter study confirmed GO‐based treatment efficacy with high MRD negativity rates in fit newly diagnosed AML patients, especially in those with low genetic risk and core binding factor, while limited benefits were observed in intermediate‐risk AML. However, further validation on larger prospective cohorts is required.

## 1 INTRODUCTION

Acute myeloid leukemia (AML) treatment has not changed for decades resulting in stagnant survival curves, as the “3 + 7” induction chemotherapy with daunorubicin and cytarabine (Ara‐C) has been the backbone regimen for fit patients.^1^ Gemtuzumab‐ozogamycin (GO), an humanized IgG4 monoclonal antibody conjugated with *N*‐acetyl‐γ calicheamicin dimethyl hydrazide, is directed against CD33, a trans‐membrane glycoprotein frequently expressed by leukemic blasts (85–90% of adult AML cases).^1^, ^2^ Calicheamicin, a natural antibiotic, has antitumor effects by producing site‐specific double‐strand DNA breaks and cell death, after CD33‐binding mediated internalization.^3^ In AML, CD33 is considered an ideal pharmacological target, because it is highly expressed on leukemic cells, while is present at very low levels on normal hematopoietic cells, thus making its targeting extremely specific for tumor cells.^4^

After first FDA approval based on early phase II trials' results, GO at a dose of 9 mg/m^2^ every 2 weeks was withdrawn in 2010, because of an exaggerated lung and liver toxicity and lethal veno‐occlusive disease (VOD) cases.^3^, ^5^, ^6^ Subsequently, several phase III trials and meta‐analysis studies have re‐evaluated efficacy and safety of GO in combination with standard induction chemotherapy using a different dose, leading to FDA approval in combination with standard induction chemotherapy. GO has been re‐approved at a new dose of 3 mg/m^2^ as first‐line treatment on Days 1, 4, and 7 and during consolidation on day 1 (up to two consolidation courses) for fit patients with de novo AML with favorable and intermediate genetic risk.^3^, ^4^, ^5^, ^6^, ^7^, ^8^, ^9^ At this reduced‐dose schedule, GO has shown a safer toxicity profile compared with first studies,^10^ and a higher survival benefit in favorable risk subjects (overall survival [OS], 20.7%) compared with intermediate AML patients (OS, 5.7%).^11^ In accordance with previously published literature, we showed also in a real‐life setting that AML patients with intermediate genetic risk might not highly benefit from the addition of GO to standard chemotherapy. Indeed, real‐life data on efficacy and safety of GO in combination with induction chemotherapy for AML treatment are still limited due to its recent approval in Italy. Therefore, we aimed at investigating efficacy and safety of GO in combination with standard chemotherapy in untreated AML patients in a real‐life Southern Italy multicenter retrospective experience.

## 2 MATERIALS AND METHODS

**FIGURE 1:**
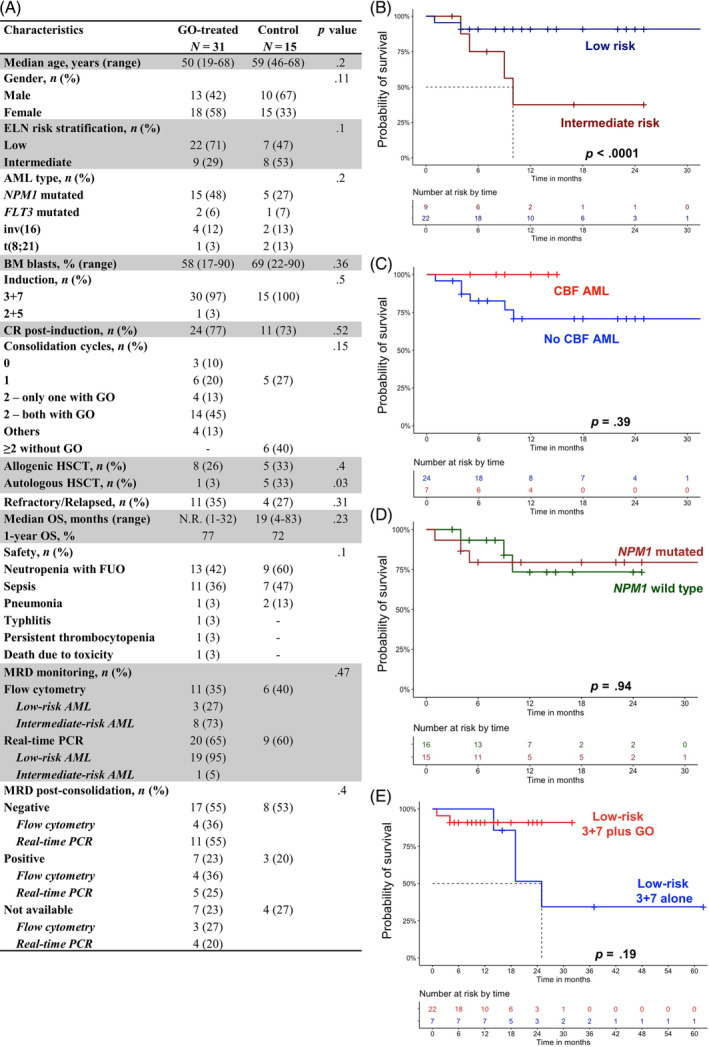
Clinical characteristics and outcomes. (A) Clinical characteristics of patients at baseline. (B) Overall survival (OS) of low (dark blue) versus intermediate (dark red) risk of acute myeloid leukemia (AML) patients in our cohort. (C) Overall survival of AML patients with core binding factor (CBF, light red) versus no CBF (blue) AML. (D) Overall survival of patients with *NPM1* mutation (bordeaux) versus patients with *NPM1* wild type (dark green). (E) Overall survival of low‐risk AML patients treated with 3 + 7 plus GO (red) or 3 + 7 alone (blue). (F) OS of intermediate‐risk AML patients treated with 3 + 7 plus GO (black). BM, bone marrow; CR, complete remission; ELN, European LeukemiaNet; FUO, fever of unknown origin; GO, gemtuzumab‐ozogamycin; HSCT, hematopoietic stem cell transplantation; MRD, minimal residual disease.

In this retrospective Southern Italy multicenter regional real‐life study, we investigated efficacy and safety of GO in newly diagnosed consecutive adult fit de novo AML patients with favorable or intermediate genetic risk, (Figure 1A), and results were compared with a control cohort treated with 3 + 7 alone, both treated from June 2020. Inclusion criteria were: age ≥18 years old; diagnosis of AML according to 2016 and 2022 World Health Organization (WHO) guidelines^12^; genetic risk stratification based on 2017 European LeukemiaNet (ELN) recommendation^13^; and at least one dose of GO at 3 mg/m^2^/dose on days 1, 4, and 7, outside clinical trials in combination with 3 + 7 induction chemotherapy, as per standard schedule (intravenous daunorubicin at 45–60 mg/m^2^ on days 1–3 and intravenous cytarabine at 100–200 mg/m^2^ on Days 1–7). GO was given only on Day 1 during consolidation at the same dosage.

Patient's fitness was assessed based on age, performance status (PS), and geriatric scales for older patients. Infusion‐related reaction prophylaxis was performed with corticosteroids, antihistamines, and acetaminophen, while tumoral lysis syndrome prophylaxis was performed with oral allopurinol and/or intravenous rasburicase. Antibiotic, antiviral, and antifungal prophylaxis was performed according to guidelines of different centers. This study was conducted in accordance with the Declaration of Helsinki, and protocols approved by our ethics committee Campania Sud, Brusciano, Naples, Italy (prot./SCCE no. 24988). All patients provided written informed consent. Primary endpoint was OS. Secondary endpoints were: rates of complete remission (CR) + CR with incomplete hematological recovery (CRi) according to 2017 ELN guidelines^13^; rate of bone marrow (BM) minimal residual disease (MRD) negativity monitored by real‐time quantitative polymerase chain reaction (PCR) or flow cytometry after the first consolidation cycle (after 28–35 days); rate of hematopoietic stem cell transplantation; and safety evaluated according to the National Cancer Institute's Common Terminology Criteria for Adverse Events version 4.0 (CTCAE v5.0).

MRD monitoring was performed on BM samples according to the 2021 ELN MRD Working Party criteria, and real‐time quantitative PCR was used for assessment of *NPM1* mutational status and chromosomal rearrangements. Flow cytometry immunophenotyping was employed for monitoring of tumor cell frequency with leukemia‐associated immunophenotypes.^14^ A *p‐*value <.01% was considered MRD negative for flow cytometry and <1000 copies/10^5^ *ABL* for real‐time quantitative PCR.^15^

### 2.1 Statistical analysis

Data were collected in spreadsheets and analyzed using R statistical software (v. 4.0.5; RStudio) and SPSS (v. 25; IBM). Differences between groups were investigated by Chi‐square, Fisher's, Wilcoxon signed‐rank, or unpaired two‐tailed *t*‐tests. Kaplan‐Meyer and log‐rank tests were adopted for survival analysis. A *p*‐value of <.05 was considered statistically significant.

## 3 RESULTS

In our study, a total of 31 consecutive patients (M/F, 13/18; median age, 50 years old) were treated with GO in combination with standard 3 + 7 chemotherapy (except one who was treated with reduced 2 + 5), and 71% of them had favorable risk, with *NPM1* the most frequently mutated gene (*N* = 15; 48%), followed by inv(16) (*N* = 4; 12%), FLT3‐ITD or ‐TKD (*N* = 2; 6%), and t(8;21) (*N* = 1; 3%). CR rate after GO‐based induction was 77%, and 45% of responders received two GO‐based consolidation cycles achieving MRD negativity in 55% of them at the end of consolidation. Autologous stem cell transplantation was possible in one subject, while eight patients were eligible to allogeneic stem cell transplant. Refractory/relapse rate was 35%, and relapse mostly occurred in the MRD positive group (*N* = 3, 43%; and *N* = 1, 6%; MRD positive and MRD negative group, respectively; *p* = .0593). Of these subjects with MRD positivity, 72% (*N* = 5) of them had low genetic risk disease, and they have received two cycles of consolidation therapy with one (*N* = 3, 43%) or two GO (*N* = 4, 57%).

Median OS of the entire population was not reached, while 1‐year OS was 77%. In particular, low genetic risk was significantly associated with increased CR rate compared with intermediate‐risk AML (88% vs. 33%, respectively; *p* < .001), as well as prolonged OS (median OS, not reached vs. 10 months; hazard ratio [HR], 0.16; 95% confidential interval [CI], 0.02–0.89; *p* < .001). Moreover, *NPM1* mutation was not correlated with better outcome (HR, 1.05; 95%CI, 0.2–5.2; *p* = .94); conversely, core binding factor (CBF) AML patients displayed a better prognosis (HR, 0.03; 95%CI, 0–87; *p* = .39; Figure 1B,C). Low‐risk AML treated with 3 + 7 plus GO showed some survival benefit compared with patients treated with 3 + 7 alone (median OS not reached vs. 25 months, respectively; *p* = 0.19), although not statistically significant because of the small sample size. Conversely, no differences were observed for intermediate‐risk AML (10 vs. 13 months, GO‐treated vs. GO‐naïve; *p* = .92; Figure 1E,F), as well as CR rates after induction (44% vs. 50%, GO‐treated vs. GO‐naïve; *p* = .5). Of note, no statistically significant differences were observed between our cohorts, except for a slightly higher rate of autologous stem cell transplantation in the control group (*p* = .03; Figure 1A). Furthermore, MRD monitoring was effectively assessed in the GO‐treated group using both real‐time PCR and flow cytometry, without statistically significant variations between methodologies in terms of MRD negativity (*p* = .3) and stem cell transplantation rates (*p* = .7), or 1‐year OS (*p* = .07; Figure 1A).

Finally, our GO‐treated patients experienced fever of unknown origin (FUO) and sepsis, with one death during induction due to septic shock, with similar rates compared with the control group (FUO, 42% vs. 60%, GO‐treated vs. GO‐naïve, *p* = .3480; and sepsis, 36% vs. 47%, GO‐treated vs. GO‐naïve, *p* = .5297). No cases of VOD occurred, even in autologous and allogeneic transplant recipients who have an increased risk of this type of complication.

## 4 DISCUSSION

Addition of GO to standard chemotherapy has been reported to be advantageous in treatment of newly diagnosed AML with intermediate‐risk cytogenetics, showing a significant improvement in response rates and lower risk of relapse.^11^, ^12^, ^13^, ^14^, ^15^, ^16^ Results from the AMLSG 09‐09 phase III trial have added evidence of clinical benefits of GO addition in *NPM1* mutant AML, while not in *FLT3* co‐mutated subjects.^17^ A recent study has confirmed efficacy and safety of GO addition to standard 3 + 7 chemotherapy also in *FLT3* mutated AML; furthermore, GO combination with midostaurin‐based induction regimens in *FLT3* mutated CD33^+^ AML has been proposed with clinical benefits and tolerability with slightly prolonged neutropenia.^11^, ^18^, ^19^ In this real‐life multicenter study from Southern Italy, our results are consistent with previously reported studies, indicating a solid benefit for low genetic risk, especially for CBF AML, although not statistically significant likely because of the small number of patients with this genetic alteration in our cohort. Conversely, intermediate‐risk patients might not highly benefit of GO in combination with standard chemotherapy as observed in low‐risk subjects, likely because of different phenotypic features making this AML subset more chemoresistant. In addition, a high MRD negativity rate was observed in responding patients, who displayed a lower AML relapse rate compared with those with MRD positivity, even though MRD monitoring was not available in 23% of cases. Indeed, GO addition to standard chemotherapy induces a high rate of MRD negativity, especially in *NPM1* mutated patients.^20^, ^21^

Our study has some limitations, including (i) the small sample size, however, to reduce single‐center physician selection on clinical outcomes and center‐specific leukemia expertise and clinical guidelines, consecutive AML patients were included from Hematology Units from all Campania region, Southern Italy; (ii) the retrospective nature of this study; and (iii) heterogeneity of MRD monitoring assessment, as our centers used single or combined methodologies. However, we confirmed similar efficacy in detecting MRD positivity for both flow cytometry and real‐time PCR, as previously described.^22^, ^23^

## 5 CONCLUSIONS

In conclusion, our real‐life multicenter study confirmed GO‐based treatment efficacy with high MRD negativity rates in fit newly diagnosed de novo AML patients, especially in those with low genetic risk and CBF. Moreover, we also confirmed a less impressive benefit of GO in intermediate‐risk AML compared with low‐risk AML, as evidenced in prior meta‐analysis. Infections might occur as a result of chemotherapy‐induced neutropenia, and require clinical management, as these complications could negatively affect outcomes. While GO addition to standard chemotherapy is unquestionably effective for low‐risk AML patients, further additional real‐life prospective studies with larger samples are needed to precisely investigate its role in intermediate‐risk AML treatment, to identify a subgroup of those patients who might greatly benefit from GO addition.

## AUTHOR CONTRIBUTIONS

*Conceptualization*: B.S. and C.S. *Clinical data*: B.S., A.P., L.A., M.A., G.D.S., D.D.N., V.G., D.M., G.S., C.C., F.G., A.M.R., and F.P. *Methodology*: D.D.N. and V.G. *Writing—original draft preparation*: B.S., D.D.N., and V.G. *Writing—review and editing*: C.S. All authors have read and agreed to the published version of the article.

## CONFLICT OF INTEREST STATEMENT

The authors declare no conflict of interest.

## ETHICS STATEMENT

Protocol approved by local ethics committee (Ethics Committee “Campania Sud,” Brusciano, Naples, Italy; prot./SCCE no. 24988).

## INFORMED CONSENT STATEMENT

Patients received informed consent obtained in accordance with the Declaration of Helsinki (World Medical Association 2013) and protocols approved by local ethics committee (Ethics Committee “Campania Sud,” Brusciano, Naples, Italy; prot./SCCE no. 24988).

## Data Availability

Data are available upon request by the authors.
